# Supplementary material for: Risk factors for severity of COVID-19 in hospital patients age 18–29 years
Source: PLoS One. 2021 Jul 30;16(7):e0255544. doi: 10.1371/journal.pone.0255544 (PMC8323903; doi:10.1371/journal.pone.0255544)
Supplement: S1 Table — (DOCX) [file pone.0255544.s001.docx]

**S1 Table. Characteristics of COVID patients aged 18-29 years, by time of severe disease or pneumonia diagnosis.**

| **Characteristics** | **Immediate** | **Delayed** | **p-value** |
| --- | --- | --- | --- |
|  | **(*n*=324)** | **(*n*=43)** |  |
| Age at encounter (years) | 26 (23-238) | 26 (22-28) | 0.56 |
| Gender |  |  | 0.15 |
| Female (not pregnant) | 173 (53.4) | 22 (51.2) |  |
| Female (pregnant) | 10 (3.1) | 4 (9.3) |  |
| Male | 141 (43.5) | 17 (39.5) |  |
| Race/Ethnicity |  |  | 0.56 |
| NH White | 52 (16.0) | 7 (16.3) |  |
| NH Black | 73 (22.5) | 10 (23.3) |  |
| NH Asian | 12 (3.7) | 0 (0.0) |  |
| NH Other Race | 2 (0.6) | 1 (2.3) |  |
| Hispanic or Latino | 173 (53.4) | 25 (58.1) |  |
| Unknown | 11 (3.4) | 0 (0.0) |  |
| Parent Hospital |  |  | 0.04 |
| Flagship hospital | 94 (29.0) | 8 (18.6) |  |
| Satellite Hospital #1 | 52 (16.0) | 4 (9.3) |  |
| Satellite Hospital #2 | 44 (13.6) | 4 (9.3) |  |
| Satellite Hospital #3 | 9 (2.8) | 5 (11.6) |  |
| Satellite Hospital #4 | 22 (6.8) | 3 (7.0) |  |
| Satellite Hospital #5 | 44 (13.6) | 5 (11.6) |  |
| Satellite Hospital #6 | 59 (18.2) | 14 (32.6) |  |
| Month of diagnostic encounter |  |  | 0.70 |
| March | 6 (1.9) | 1 (2.3) |  |
| April | 13 (4.0) | 1 (2.3) |  |
| May | 14 (4.3) | 2 (4.7) |  |
| June | 101 (31.2) | 12 (27.9) |  |
| July | 98 (30.2) | 12 (27.9) |  |
| August | 23 (7.1) | 2 (4.7) |  |
| September | 16 (4.9) | 5 (11.6) |  |
| October | 22 (6.8) | 3 (7.0) |  |
| November | 26 (8.0) | 3 (7.0) |  |
| December | 5 (1.5) | 2 (4.7) |  |
| Social Vulnerability Index |  |  | 0.70 |
| <20th percentile | 49 (15.1) | 7 (16.3) |  |
| 20-39th percentile | 55 (17.0) | 11 (25.6) |  |
| 40-59th percentile | 65 (20.1) | 9 (20.9) |  |
| 60-79th percentile | 75 (23.1) | 9 (20.9) |  |
| 80-99th percentile | 63 (19.4) | 5 (11.6) |  |
| Missing | 17 (5.2) | 2 (4.7) |  |
| Financial Class |  |  | 0.93 |
| Private insurance | 143 (44.1) | 20 (46.5) |  |
| Medicare/Medicaid | 55 (17.0) | 8 (18.6) |  |
| Self-Pay | 124 (38.3) | 15 (34.9) |  |
| Other | 2 (0.6) | 0 (0.0) |  |
| BMI (kg/m^2^), categorical |  |  | 0.88 |
| Underweight ≤18.5 | 4 (1.2) | 0 (0.0) |  |
| Normal Weight 18.5-25 | 35 (10.8) | 4 (9.3) |  |
| Overweight 25-30 | 60 (18.5) | 5 (11.6) |  |
| Class 1 Obesity 30-35 | 69 (21.3) | 12 (27.9) |  |
| Class 2 Obesity 35-40 | 49 (15.1) | 6 (14.0) |  |
| Class 3 Obesity >40 | 80 (24.7) | 13 (30.2) |  |
| Missing | 27 (8.3) | 3 (7.0) |  |
| Exposure History |  |  | 0.10 |
| No known exposure | 128 (39.5) | 16 (37.2) |  |
| Any known exposure | 41 (12.7) | 5 (11.6) |  |
| Known HH exposure | 70 (21.6) | 16 (37.2) |  |
| Missing | 85 (26.2) | 6 (14.0) |  |
| Symptom Screen |  |  | 0.03 |
| Negative | 71 (21.9) | 10 (23.3) |  |
| Positive | 138 (42.6) | 26 (60.5) |  |
| Missing | 115 (35.5) | 7 (16.3) |  |
| Charlson Comorbidity Index Score |  |  | 0.32 |
| 0 | 215 (66.4) | 23 (53.5) |  |
| 1 to 2 | 84 (25.9) | 16 (37.2) |  |
| 3 to 4 | 13 (4.0) | 2 (4.7) |  |
| > 4 | 12 (3.7) | 2 (4.7) |  |
| Admission Category |  |  | <0.001 |
| Emergency | 123 (38.0) | 41 (95.3) |  |
| Inpatient | 189 (58.3) | 1 (2.3) |  |
| Observation | 12 (3.7) | 1 (2.3) |  |
| Other | 0 (0.0) | 0 (0.0) |  |

IQR: Interquartile range. NH: Non-Hispanic. BMI: Body mass index.
