# Supplementary material for: Risk factors for severity of COVID-19 in hospital patients age 18–29 years
Source: PLoS One. 2021 Jul 30;16(7):e0255544. doi: 10.1371/journal.pone.0255544 (PMC8323903; doi:10.1371/journal.pone.0255544)
Supplement: Attachment — Submitted filename: Response to Reviewers.pdf [file pone.0255544.s002.pdf]

PONE-D-21-13036
Risk Factors for Severity of COVID-19 in Hospital Patients Age 18-29 Years
PLOS ONE

Journal requirements:

When submitting your revision, we need you to address these additional requirements.
1. Please ensure that your manuscript meets PLOS ONE's style requirements, including those for file naming.

Response: We have revised the manuscript and file names to meet the PLOS ONE's style requirements.

2. Data Availability

Response: Data cannot be shared publicly because of patient confidentiality concerns as imposed by the Houston Methodist Institutional Review Board. Access to de-identified data can be made to Jennifer Meeks (jmeeks@houstonmethodist.org) which will be evaluated on a case by case basis in line with institutional policies.

3. We note that Figure 3 in your submission contain map images which may be copyrighted.

Response: We have removed the copyrighted base map in the revised Figure 3.

4. Please include captions for your Supporting Information files at the end of your manuscript, and update any in-text citations to match accordingly. Please see our Supporting Information guidelines for more information: http://journals.plos.org/plosone/s/supporting-information.

Response: We have included the captions for the Supporting Information files at the end of the manuscript and updated the in-text citations accordingly.

RESPONSES TO THE REVIEWER'S COMMENTS

We thank the reviewers for their insightful comments. Please find below our detailed point-by-point response (in blue) to each of the reviewers' comments/recommendations with the line numbers indicating location of the changes in the clean version of the manuscript.

Reviewer #1: Sandoval et al describe risk factors associated with severe COVID-19 in patients aged 18-29 years seen at a large hospital system in Texas.

General comments:
- Females who are pregnant during the pandemic visit the hospital because of regular check-ups

of their pregnancy or labor. These hospital visits are unrelated to COVID-19 however these women were regularly screened for COVID-19 to prevent the spread of the disease. This group of COVID-19 positive patients is a particular group of COVID-19 patients, which may need to be highlighted more in the discussion. I am not sure if I agree with the author's statement that pregnant women provide insight into disease dynamics of the general population. This only female group has different healthcare utilization needs than the general population.

Response: Thank you for the thoughtful review. We agree that pregnant patients represent a distinct source population in terms of COVID-9 screening and healthcare utilization. We have removed the statement "indeed, pregnant women presenting for routine prenatal care or labor and delivery could provide insight into disease dynamics in the general population" and emphasized the issue more in the discussion as follows:

"Of note, pregnant women were far less likely to be diagnosed with pneumonia or other disease indicators than either non-pregnant women or men, possibly due to being regularly screened for COVID-19 during their routine prenatal or labor visits which are unrelated to COVID-19. Pregnant patients may therefore represent a population of largely subclinical COVID-19 cases who were diagnosed incidentally, and future studies are needed to investigate long-term maternal and fetal outcomes of symptomatic and asymptomatic patients." (lines 319-324)

- Throughout the abstract and manuscript, the authors report the OR / aOR with the p-value in the text. I have a preference for reporting the 95% confidence interval because the range of the confidence interval provides additional information over the p-value.

Response: We have added the 95% CI confidence intervals to the reported OR / aOR in the revised manuscript.

- The authors were only able to catch diagnosis/ readmissions within their 30-day time frame if patients presented again to one of the hospitals in the health system. The authors need to acknowledge that it may be possible that patients presented elsewhere which affects their outcomes.

Response: Thank you for your comment. We have added the following sentences to the limitations to address the issue:

"Finally, our findings may underestimate the actual 30-day outcomes because we cannot rule out the possibility that, following their initial encounter, patients sought further care at an institution outside of the Houston Methodist Hospital System, where the outcome data are not available to the research team." (lines 393-395)

- How did the authors decide on whether these diagnoses were COVID-19 related and therefore COVID-19 severe disease? It could of course very well be that a patient had a myocardial infarction unrelated to COVID-19 in the 30 days following a COVID-19 diagnosis?

Response: Given the distinctions between the diagnoses that are related versus unrelated to COVID-19 were not well defined in the EMR, the composite disease outcomes in our analysis

were defined as ‘all-cause’ outcomes. We have added the following sentence in the limitations to clarify the issue:

“Given the distinctions between the diagnoses that are related versus unrelated to COVID-19 were not well defined in the EMR, the composite disease outcomes in our analysis were defined as ‘all-cause’ outcomes. Therefore, our findings may overestimate the actual COVID-19 related outcomes. Despite the limitations, our study is one of few studies reported the important longitudinal health consequences in young COVID-19 patients, at both inpatient and emergency department encounters.” (lines 396-400)

Abstract:
- The sentence: “This study was limited to young adults diagnosed at a hospital encounter and results may not be generalizable to all COVID-19 patients” can be removed from the abstract as it is clear from the research question that this study focused solely on young adults.
Response: Thank you for your comment. We have removed the sentence “This study was limited to young adults diagnosed at a hospital encounter and results may not be generalizable to all COVID-19 patients” from the Methods and Findings subsection of the Abstract.

Introduction:
- The authors mention that few (two) studies included a young patient population. Please include their findings in the introduction and explain why current study is different. This information may be more elaborately included in the discussion.
Response: Thank you for your comments. We have added the following sentences to the Introduction as recommended by the reviewer. In our original Discussion section, we had discussed these studies as below.

Introduction:
“However, these studies were either conducted with a small sample size [6,8] or they only reported preliminary data on the proportion of patients who experienced the composite event of death and mechanical ventilation [7].” (lines 71-73)

Discussion:
“The previously published research articles describing risk factors for severe COVID-19 in young adults included a combined total of fewer than 1,500 patients, and primarily included information from the diagnostic encounter [6-8]. Our findings constitute a substantial addition to the existing knowledge base because we not only included data for young adults diagnosed at both inpatient and emergency department encounters, but also collected longitudinal outcome data, thus allowing us to characterize patients during stages of disease progression.” (lines 381-386)

Methods
- I suggest to move the paragraph regarding geographic data collection and analyses to the end of the methods (just before the role of the funding source.

Response: We have moved the paragraph regarding geographic data collection and analyses to the end of the Methods section as recommended by the reviewer.

- How did the authors finalize the list of diagnoses that defined severe disease?
Response: In our analysis, the list of the diagnoses that defined the composite disease outcomes were determined based on the severity described in the current literature and the frequency of the disease identified in the population. The list of diagnoses was finalized in consultation with experienced clinicians. We have updated the Methods accordingly.

"Diagnoses that defined the composite disease outcomes were determined based on the severity described in current literature and the frequency of the disease identified in the population. The list of diagnoses was finalized in consultation with experienced clinicians" (lines 129-132)

Results:
- Table 1: Please explain the area deprivation index in the methods; what does the scale 1 through 10 represent?

Response: We have added the following sentence to the Methods to clarify the issue:

"The Area Deprivation Index which measures relative deprivation amongst all census block groups in the state of Texas on a scale of 1-10, where one (1) is the least disadvantaged and ten (10) is the most disadvantaged". (lines 173-175)

- Given that this a young study population and you would expect patients to be relatively healthy, would it not be better to have the cut-off values for the Charlson comorbidity index be: 0, 1, 2, > 2 or even 0, 1, >1 as most patients (77.6%) do not have a comorbidity? (Table 1)

Response: We agree this population has a relatively low Charlson comorbidity index (CCI) compared to the general population. Because the CCI is collinear with the component medical and surgical history factors, it was not included in regression analyses. Therefore, we felt it was important to characterize our relatively healthy young adult cohort using the full CCI in Table 1, to facilitate comparison to the general population.

- In the text, the authors mention that 43% of the patients reported cough, sore throat and/ or shortness of breath, while table 2 refers to these symptoms as respiratory symptoms. Can the authors describe which symptoms were included in each group of symptoms mentioned in table 2?

Response: We have added the following footnotes to Table 2 to clarify the components of each group of symptoms:

“Systemic symptoms included fever, chills, myalgias, arthralgias, fatigue, and malaise. Respiratory symptoms included cough, shortness of breath, and sore throat. Neurologic symptoms included loss of smell or taste, and headache. GI symptoms included nausea, vomiting, diarrhea, and cramping.” (Table 2’s footnotes)

- What is the relevance of being hospitalized to a satellite hospital compared to the flagship hospital?

Response: Thank you for your comment. We have added the following paragraph to the Discussion to address the relevant of the issue raised by the reviewer:

“Although the adverse outcomes in COVID-19 patients may also be affected by certain hospital-related factors (such as catchment population, staff experience, referral or community hospital, and equipment capacity), published data on this issue appears to be unavailable. Therefore, we attempted to address the issue by evaluating the hospitalization to a satellite hospital versus the flagship hospital is relevant and warrants continued investigation. Our findings of having increased number of patients with composite disease outcomes in patients hospitalized presenting to our flagship hospital is consistent with the fact that our flagship hospital is a tertiary hospital located in a large medical center and received more severe referrals needing higher levels of care than satellites hospitals, especially early in the outbreak. Additionally, the flagship hospital is centrally located and serves high population-density, urban communities surrounding the medical center. Meanwhile, given the higher odds of subsequent hospital encounter within 30 days among patients who initially presented to the in the satellite hospitals compared to the flagship hospital, we could not rule out the contribution of the staff experience level and equipment capacity. Further studies on this issue would be programmatically appropriate.” (lines 364-377)

- Can the authors explain why “missing BMI” is relevant to describe as a protective factor for readmission?

Response: Thank you for your comment. We have added the following sentence to the Discussion to discuss the issue:

“Of note, our analysis found that patients with a missing BMI had lower odds of readmission compared with patients having a normal BMI. In fact, the small group of missing BMI included patients having much fewer underlying conditions with most of the patients having CCI ≤ 2. Given the small sample size of the missing BMI category, we could not rule out the possibility that those patients actually had normal BMIs and the significance seen in the readmission difference with the current normal BMI group occurred by chance.” (lines 342-348)

- Can the authors explain the following inconsistencies in tables 4 through 6:
o Gender has a reference group in the univariable analysis and not in the multivariable analysis

Response: We have added the note indicating the reference group for gender in the multivariable analysis in Tables 4-6.

o What is the meaning of the ** for certain covariates? Why are there no results for certain categories (for instance for: NH Asian, Satellite Hospital #3, certain months, any known exposure.. etc.) Seen the relatively low number of patients per covariate group, the authors should consider regrouping the covariates.

Response: Thank you for your comment. Although all the variables were evaluated in the univariable analysis, not all of them went through the variable selection process to be included in the final multivariable model (e.g. some binary variables in the medical history). For certain categorical variables, such as race/ethnicity and Social Vulnerability Index, the adjusted OR for some categories cannot be generated due to the low number of patients. We have revised the Methods to clarify the variable selection process as follows:

“The selection of variables for the multivariable models were conducted using the least absolute shrinkage and selection operator (LASSO) method with the cross-validation selection option and clinical importance of the covariates [27]. Briefly, all variables used in the univariable analysis were assessed by the LASSO program, which suggested good models that included the variables with the highest probability of being a risk factor. During the modeling process, the potential risk factors were discussed with senior clinicians who have extensive clinical experience in the field to ensure the biological plausibility of the selected covariates. To avoid over-fitting, some variables which were significant in the univariate analysis, but insignificant in multivariable modeling were not selected in the final model if their exclusion did not affect the diagnostic performance of the final model which was determined by a non-significant likelihood ratio test result and the area under the Receiver Operating Characteristic (ROC) curve.” (lines 153-164)

o I assume that the authors calculated the OR and aOR separately per medical history and surgical history. Can the authors explain why only the OR is reported for cholecystectomy (which is mentioned be the authors when describing table 1), while the aOR is reported for solid organ transplant (with a wide 95% confidence interval).
Response: Each component of the medical history and surgical history was calculated as a separate, binary variable. As mentioned in the response right above, only variables selected passed through our variable selection process using the combination of the clinical importance and LASSO variable selection were included in the final multivariable model.

- The following sentence is contradictory and the word likewise does not seem to be correct as non-Hispanic Black patients were not more likely to be diagnosed with severe disease or pneumonia while Hispanic patients were, please revise: “Notably, non-Hispanic Black patients were not more likely to be diagnosed with severe disease or pneumonia, but they were likely to return to the hospital within thirty days, compared to White patients; likewise, Hispanic patients were more likely to be diagnosed with severe disease or pneumonia compared to White patients”

Response: Thank you for your comments. We have revised the sentences as recommended by the reviewer:

“Notably, non-Hispanic Black patients were not more likely to be diagnosed with composite disease outcomes or pneumonia, but they were likely to return to the hospital within thirty (30) days, compared to White patients; in contrast, Hispanic patients were more likely to be diagnosed with composite disease outcomes or pneumonia compared to White patients”. (lines 330-334)

- Given the amount of information provided by the authors in the results, I find the discussion relatively short and lacking details/ in depth descriptions of the findings and comparison with already published manuscripts. Topics of interest may include: pregnancy, results of other studies that included younger patient populations, information of marginalized populations (as this is highlighted in the abstract), etc.

Response: Thank you for your comments. We have expended the Discussion as per the reviewer’s recommendations.

References:
- Please update references when more details about recently published manuscripts are currently available (e.g. Cunningham JW, Vaduganathan M, Claggett BL, Jering KS, Bhatt AS, Rosenthal N, et al. Clinical Outcomes in Young US Adults Hospitalized With COVID-19)

Response: Thank you for your comment. We have updated the references in the revised manuscript.

Reviewer #2: Abstract/Methods: Please explain the acronym CHF

Response: Thank you for your comment. Congestive heart failure has been written out in the Abstract.

Introduction: 1. 'were young adults, aged 18-29 years,.' : no coma before full-stop.

Response: Thank you for identifying the typo. We have removed the redundant comma in the revised manuscript.

Methods: Please explain , what do you mean "Patients were included if they received a positive diagnostic result from a severe acute respiratory syndrome coronavirus 2 (SARS-CoV-2) RNA polymerase chain reaction (PCR) assay".is there a PCR assay for a severe acute respiratory syndrome SARS-CoV-2?

Response: We have revised the sentence as follows to clarify the issue:

“Patients were included if they received a positive diagnostic result associated with a hospital encounter from either (1) An RNA polymerase chain reaction (PCR) test for the severe acute respiratory syndrome coronavirus 2 (SARS-CoV-2) or (2) a SARS-CoV-2 antigen test.” (lines 101-104)

Methods/ Electronic Medical Record Data Collection:The authors state that" BMI was calculated and classified according to the Centers for Disease Control and Prevention (CDC) and World guidelines Health Organization (WHO)" You provide the same reference for CDC and WHO as well.

Response: Thank you for your comment. We have replaced the mentioned reference by the one from the CDC and accordingly updated the citation in the text as follows:

“BMI was calculated and classified according to the United States Centers for Disease Control and Prevention (CDC) guidelines [14].” (lines 114-117)

Reviewer #3: To the authors
The current manuscript is interesting and important. The authors found some factors associated with severity in young COVID-19 patients. The current manuscript involves several factors associated with short- and long-term health consequences of this emerging infectious disease in young adults

Response: Thank you for your insightful comments and enthusiasm.

My questions:
Methods
+Acute kidney injury is associated with poor prognosis in COVID-19 patients. I have not found how many patients had AKI. Do you have any data about AKI in those patients?

Response: Thank you for your comment. We agree that acute kidney injury is an important outcome among COVID-19 patients, and we have updated the composite disease outcome table (Table 3) to include AKI. We found three (3) patients with incident AKI following a positive COVID-19 diagnostic test, and updated Table 3 accordingly. All three (3) of the patients with incident AKI within 30 days of initial encounter also had at least one other diagnosis from the composite definition, so the regression models were unaffected.

+I have found no reports of the use of psychoactive drugs. Do you have any record about psychiatric disease and drug users? Is there any relation between severe COVID-19 and both psychiatric disease and drug users?

Response: Thank you for your comment. While we were unable to collect any data on psychiatric drug use, we did include history of documented mental illness as a component in regression analyses.

Discussion
+In my opinion, you should write about the therapies used, such as azithromycin and dexamethasone, used in these patients in the discussion.

Response: We have added information about therapies received in the discussion as follows: "While relatively few patients received drugs such as dexamethasone or remdesivir, azithromycin treatment in the initial encounter was a risk factor for returning to the hospital." (lines 328-329)

Reviewer #4: Sandoval et al. conducted a retrospective observational study of n=1853 young adults (18–29 years) seen in the ED or admitted to hospital at the time of a positive respiratory swab for SARS-CoV-2 in a single healthcare system in Houston, TX. Using logistic regression models, they evaluated risk factors for pneumonia, "other severe disease outcomes," and hospital admission or readmission within 30 days. Thank you for the opportunity to review this manuscript.

Response: Thank you for your thorough and thoughtful review.

1. Please provide more details about the initial hospital encounters. For example, what proportion of these were ED visits only? What proportion of individuals were initially hospitalized? Did the associations reported differ across these strata?

Response: We have revised Tables 1 and 2 to clarify the issue. Admission type (ED, inpatient, etc) is included in Table 1 (overview) and in the analysis of risk factors for subsequent hospital encounters. Emergency department encounters not associated with an inpatient admission were classified as 'emergency department only', while ED encounters leading to an inpatient admission (transferred to a floor) were collapsed and classified as inpatient admissions.

2. Some terminology used here is difficult. To call the original hospital encounter an "admission" may be unclear to some readers. I would suggest referring to this as a "hospital encounter."

Response: Thank you for your comment. We have replaced "admission" by the "hospital encounter" through the manuscript where it was appropriate.

3. In addition, the term “readmission” could include a hospital readmission for individuals who were originally admitted or a first admission for individuals who were seen in the ED. I think it would be more clear to call this outcome a “second hospital encounter” and not a readmission.

Response: Transfers and linked admissions, ie ED to inpatient admissions were not included in subsequent hospital encounters definitions. Return to hospital analyses were limited to patients who were discharged home from their initial encounter. In the revised manuscript, we have replaced the “readmission” to the “subsequent hospital encounter” or “return to hospital”.

4. Similarly, the outcome described as “severe disease” is also confusing. In COVID-19, the CDC defines severe outcomes as hospitalization, ICU admission, intubation, and death, but here the authors have something different in mind. This composite outcome should be described in a different way. Alternatively, consider using a more standard endpoint, such as ICU admission or mechanical ventilation.
Response: Thank you for your comment. We have replaced the “severe disease” by the “composite disease outcome” and clarify the definition in the Methods.

5. Why were pregnant individuals excluded from some analyses?

Response: Thank you for your comment. We excluded pregnant patients from the return to hospital analyses because we expect pregnant patients to return to the hospital for prenatal and labor and delivery encounters unrelated to COVID-19. We have added the following sentence to the Methods: to clarify the issue.

“Pregnant patients were excluded from 30-day repeat hospital encounters analyses, as their healthcare utilization differs significantly from the general population.” (lines 143-144)

6. Please include the covariates that were considered in the variable selection step. How were variables discarded?

Response: As mentioned in the response above to a comment from reviewer #1, all variables used in the univariable analysis were evaluated in the variable selection process based on the clinical importance and LASSO variable selection. We have added the following paragraph to the Methods to clarify the variable selection process:

“The selection of variables for the multivariable models were conducted using the least absolute shrinkage and selection operator (LASSO) method with the cross-validation selection option and clinical importance of the covariates [27]. Briefly, all variables used in the univariable analysis were assessed by the LASSO program, which suggested good models that included the variables with the highest probability of being a risk factor. During the modeling process, the potential risk

factors were discussed with senior clinicians who have extensive clinical experience in the field to ensure the biological plausibility of the selected covariates. To avoid over-fitting, some variables which were significant in the univariate analysis, but insignificant in multivariable modeling were not selected in the final model if their exclusion did not affect the diagnostic performance of the final model which was determined by a non-significant likelihood ratio test result and the area under the Receiver Operating Characteristic (ROC) curve." (lines 153-164)

Minor comments

The authors write, "(few previous studies) have incorporated longitudinal clinical data." For clarity, I'd suggested rephrasing this to state, "Among studies in young adults, few (or none?) have incorporated longitudinal clinical data."

Response: Thank you for your suggestion. We have revised the mentioned sentence as follows: "Among studies in young adults, only a few have incorporated longitudinal clinical data." (lines 70-71)
